# A Deep Reinforcement Learning Strategy for Surrounding Vehicles-Based Lane-Keeping Control

**DOI:** 10.3390/s23249843

**Published:** 2023-12-15

**Authors:** Jihun Kim, Sanghoon Park, Jeesu Kim, Jinwoo Yoo

**Affiliations:** 1 Graduate School of Automotive Engineering, Kookmin University, Seoul 02707, Republic of Korea; wkdrns3847@gmail.com (J.K.); ppp7326@naver.com (S.P.); 2 Departments of Cogno-Mechatronics Engineering and Optics and Mechatronics Engineering, Pusan National University, Busan 46241, Republic of Korea; jeesukim@pusan.ac.kr; 3 Department of Automobile and IT Convergence, Kookmin University, Seoul 02707, Republic of Korea

**Keywords:** reinforcement learning, autonomous vehicles, advanced driver assistance, vehicle control, safety

## Abstract

As autonomous vehicles (AVs) are advancing to higher levels of autonomy and performance, the associated technologies are becoming increasingly diverse. Lane-keeping systems (LKS), corresponding to a key functionality of AVs, considerably enhance driver convenience. With drivers increasingly relying on autonomous driving technologies, the importance of safety features, such as fail-safe mechanisms in the event of sensor failures, has gained prominence. Therefore, this paper proposes a reinforcement learning (RL) control method for lane-keeping, which uses surrounding object information derived through LiDAR sensors instead of camera sensors for LKS. This approach uses surrounding vehicle and object information as observations for the RL framework to maintain the vehicle’s current lane. The learning environment is established by integrating simulation tools, such as IPG CarMaker, which incorporates vehicle dynamics, and MATLAB Simulink for data analysis and RL model creation. To further validate the applicability of the LiDAR sensor data in real-world settings, Gaussian noise is introduced in the virtual simulation environment to mimic sensor noise in actual operational conditions.

## 1. Introduction

With the emergence and development of deep learning frameworks, vehicle autonomous driving technologies have undergone rapid advancement in recent years, especially in object recognition and decision-making [1,2]. Interest in autonomous driving technologies, driven by the 2004 DARPA Challenge, has grown with the concerted efforts of original equipment manufacturers (OEMs) and industry stakeholders [3,4]. With these technological advancements, various U.S. states and the federal government have been preparing regulations and guidelines for the introduction of autonomous vehicles (AVs) [5]. Additionally, functional safety standards such as ISO 26262 [6] have been established. The development of autonomous driving technologies is expected to help address various road-related issues such as safety, traffic congestion, energy efficiency, and environmental impact [7,8]. However, the continued advancement of these technology encounters numerous challenges.

Lane-keeping systems (LKS), a key feature of advanced driver assistance systems (ADAS), allows vehicles to safely maintain their lanes [3]. However, most LKS rely heavily on camera sensors. Therefore, in situations where lane recognition involves uncertainties related to factors such as sensor damage, the system performance deteriorates, potentially leading to accidents [9]. The United Nations Economic Commission for Europe WP.29 [10], an international regulatory body for automotive standards, has established various regulations for AVs through its subsidiary, the United Nations Economic Commission for Europe (GRVA UNECE), specifying the minimal risk maneuver (MRM) functionality that LKS must incorporate [11]. This functionality emphasizes the importance of maintaining a stable lateral position in hazardous situations to avoid disrupting traffic flow, highlighting its critical role as an essential safety feature in AVs. Autonomous driving technologies classified at Level 3 or higher are designed to issue a takeover request (TOR) to the driver in the event of hazardous situations, such as sensor failures or system limitations within the perception system [12]. If the driver fails to respond and take control, the system is required to perform MRM [13,14].

To address these challenges, we propose a solution based on the deep deterministic policy gradient (DDPG) reinforcement learning (RL) model [15]. While previous implementations of RL in autonomous driving technologies were focused on path planning or trajectory tracking [16,17], in this study, we incorporate LKS by considering the presence of surrounding static and dynamic objects, using information obtained from LiDAR sensors. LiDAR sensors exhibit exceptional precision in gauging distances, velocities, and directional information in the proximity of a vehicle. Their efficacy remains robust even under challenging low-visibility conditions, such as those posed by fog or dust. This approach allows the AV to navigate safely, even when the camera sensor is not functioning. The use of the DDPG model for lane-keeping control is expected to significantly enhance safety by allowing an AV to maintain its lane securely while responding to hazardous situations, thereby preventing traffic accidents.

**Figure 1:**
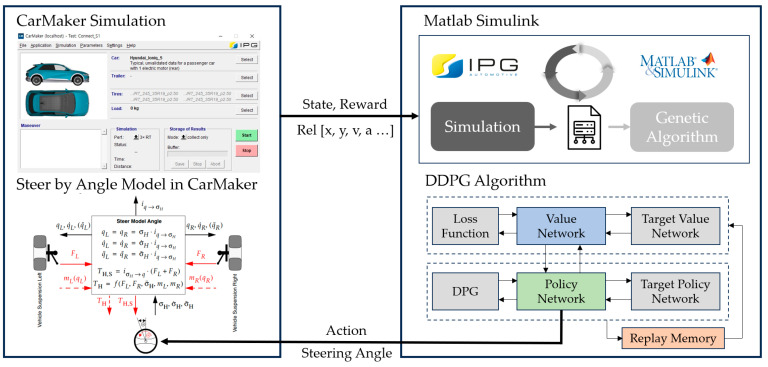
CarMaker and MATLAB Simulink integrated environment.

In summary, this paper proposes an LKS control method using DDPG RL, based on the movements of surrounding vehicles when lane recognition is compromised due to degraded camera sensor performance. As illustrated in Figure 1, learning is implemented in an integrated environment, combining the simulation program IPG CarMaker and MATLAB Simulink. Within the simulation environment of CarMaker, essential data pertaining to surrounding objects and the behavior of the vehicle is generated. This state information is subsequently transmitted to the Matlab Simulink environment. Following this data exchange, the steering control values, which are obtained through reinforcement learning, are then employed to govern the vehicle within the CarMaker environment. MATLAB Simulink is interconnected with CarMaker in real-time, enabling monitoring of control inputs and the vehicle outputs. Furthermore, it provides the Reinforcement Learning Toolbox, supporting reinforcement learning algorithms like DQN, PPO, and DDPG. This toolbox facilitates efficient development through hyperparameter configuration and training progress monitoring.

**Figure 2:**
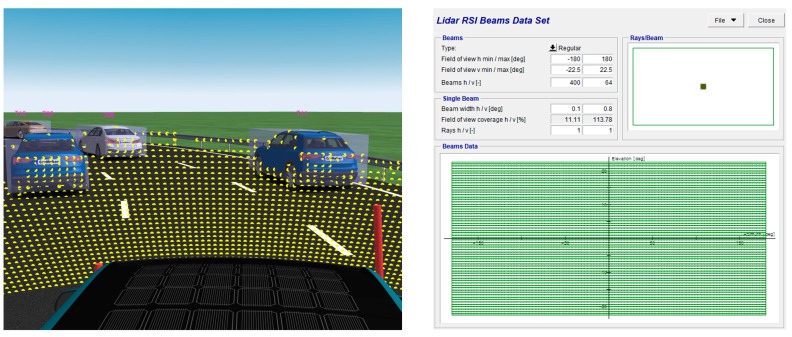
CarMaker LiDAR sensor data visualization and RSI data set.

The LiDAR sensor was set up based on the specifications of the Ouster OS1-64. The OS1-64 LiDAR sensor actually boasts a vertical resolution of 64, a horizontal resolution of 1024, and a field of view ranging from 360 degrees horizontally and −22.5 degrees to 22.5 degrees vertically, as shown in Figure 2. The study does not use deep learning models for recognition. Instead, the state information provided to the reinforcement learning model is obtained through dynamic calculations within the simulation. However, to mimic the operational conditions of sensors installed in real vehicles and validate the applicability of the approach in real-world scenarios, we introduce Gaussian noise into the system [18].

The methodology employed in the research is an end-to-end reinforcement learning (RL) approach, which utilizes information, including the relative positions, relative velocities, and relative heading angles of surrounding vehicles, to generate the steering angle. The steering angle generated by the agent is used to control the vehicle through a PID controller. The proposed approach is evaluated through a comparison with traditional control methods, including the pure pursuit and Stanley control methods.

## 2. Related Work

### 2.1. Traditional Lateral Controller for Autonomous Vehicle

In traditional autonomous driving systems, lane information is typically obtained through cameras or vehicle position data derived from HD maps. This information is then used to generate driving paths, followed by trajectory control [19]. Path-tracking control techniques include controllers that track the path generated from the vehicle position and can be categorized into kinematic-based, dynamic-model-based, and feedback-based approaches [20]. In conventional control methods, steering angles are computed based on lane information. The representative traditional control systems include the pure pursuit controller and the Stanley controller. The pure pursuit controller is a path-tracking controller based on geometric methods, which offers stability against disturbances. It delivers excellent performance in terms of ride comfort and tracking capability and can be tuned to achieve superior results, rendering it a popular choice for testing in many AVs [21,22]. The Stanley controller, which emerged during the 2005 DARPA Grand Challenge [3,4], controls the vehicle by focusing on the point closest to the target point from the vehicle’s front axle. In this study, we control the vehicle using steering angle values through RL in the absence of lane information and compare its performance with conventional control methods such as pure pursuit and Stanley controllers. The performance of the pure pursuit and Stanley controllers was effectively optimized through the parameter tuning process, considering road and driving speed conditions specific to the operational design domain (ODD) for the experiment.

### 2.2. Autonomous Fault-Tolerant System

As autonomous driving technology advances, the role of the traditional driver is transitioning to autonomous driving systems. However, ensuring safety in the complex and highly variable road driving environment remains a challenging task. AVs rely on a variety of sensors, which increases the likelihood of issues arising due to physical or electrical sensor failures [23]. Creating autonomous vehicles that are immune to sensor failures is impossible; therefore, the development of a fault-tolerant system (FTS) for autonomous vehicles is necessary to ensure safety even in the event of failures. The FTS starts with the detection of faulty sensors in the autonomous driving system. Realpe and Vintimilla [24] proposed a fault-tolerant perception paradigm for the sensor suite of autonomous driving systems. The paradigm presented in their paper involves detecting failures in vision sensors used for perception and outlines a data fusion architecture to minimize risks. Rather than relying on a single sensor, it employs a multi-sensor-based vehicle architecture for fault detection and diagnosis, combining and diagnosing sensor data. This approach allows for higher reliability and safety. After detecting faults in the system, fault-tolerant techniques become necessary. Kans and Lee [25] introduced a fault-tolerant control method for lane-keeping systems (LKS) to address situations where lane recognition becomes impossible due to a camera sensor failure. In the event of a camera sensor failure, they create a virtual lane using a lateral vehicle model, GPS, and in-vehicle sensors, enabling the vehicle to maintain lateral control performance for up to 3 s and minimize risks. Existing FTS solutions for LKS have proven effective in ensuring safety for short durations. In this study, we assume a camera sensor failure in a camera-based LKS and aim to implement FTS functionality using LiDAR sensor-based reinforcement learning.

### 2.3. Application of Deep Learning Technology in AVs

Artificial intelligence technology is being employed across various industries, thanks to the rapid advancement of deep learning algorithms and the improved performance of GPU hardware for computations. The field of autonomous driving is no exception, as it prominently demonstrates the application of deep learning technology [26]. Companies like Tesla in the United States have openly shared a variety of deep learning techniques and architectures they are currently utilizing. Consequently, there is an ongoing effort to collect and make available the datasets required for deep learning training in autonomous driving. Furthermore, organizations such as Waymo and Argoverse have been actively collecting and sharing diverse sets of training data, fostering vibrant research in this domain. AVs execute perception, decision-making, and control based on an array of sensors, offering multiple avenues for the application of deep learning technology [27]. The shift from traditional rule-based approaches to artificial intelligence technology allows for more adaptive responses even in previously unpredictable scenarios.

#### 2.3.1. Object Detection Using Deep Learning

Cognitive technology is the technique of detecting vehicles, obstacles, and more from various sensors such as cameras, LiDAR, ultrasonic, and radar, primarily focusing on research involving camera and LiDAR sensors. Camera-based cognitive deep learning models, such as YOLO, Faster R-CNN, and SSD, offer excellent object classification based on high-resolution images but are sensitive to environmental changes and have limitations in estimating 3D information accurately from 2D images [28]. In contrast, LiDAR recognition technology provides accurate 3D information, even during nighttime, based on precise distance data. LiDAR-based recognition models like VoxleNet, PointRCNN, and PV-RCNN exhibit high performance in 3D distance information [29]. This implies that LiDAR recognition technology holds promise for enhancing the safety of autonomous vehicles.

#### 2.3.2. Vehicle Control Technology Using RL

RL is a subfield of machine learning that aims to determine an optimal strategy through a series of decision-making processes. This approach has been rapidly developing in recent years and is being applied to complex systems such as long-distance vehicle control. In the field of autonomous drones and vehicles, research on control mechanisms based on hybrid RL is expanding to reduce the need for specific designs for new tasks. Off-policy actor–critic methods have demonstrated performance in continuous control tasks. Representative algorithms include DDPG, TD3, and SAC [30]. In the field of vehicle lateral control and decision-making, many studies have used the DDPG algorithm. Moreover, RL has been applied to actual vehicle lateral control [16]. These applications involve following a path defined by waypoints spaced at intervals of 5 m, with continuous updates associated with the Euclidean distance, angle deviation, and perception module data, which are provided as inputs to the RL agent. Furthermore, the integration of dynamic properties into the reward components of RL has proven effective in addressing vehicle safety while effectively executing lateral control [31]. The DDPG algorithm is relatively easy to understand, intuitive, and relatively simple to implement and debug compared to other algorithms. In this study, we also used the DDPG algorithm because the values used as reinforcement learning data are not high-dimensional but are continuous real-valued observations, and the control extracts the steering angle as the result. In addition, when a comparative experiment was conducted with TD3, the learning speed was faster and demonstrated similar performance upon reaching a steady state.

## 3. LKS Method Based on DDPG

### 3.1. Proposed Method for LKS Using RL

**Figure 3:**
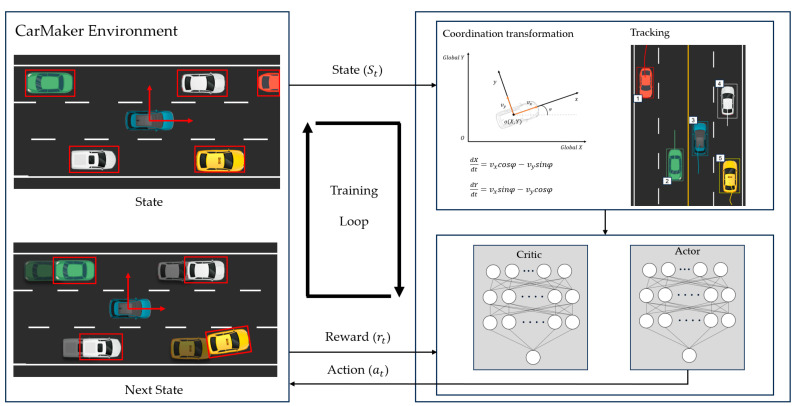
Reinforcement learning methodology and learning architecture for LKS.

This paper proposes an RL-based solution for LKS, using LiDAR sensor data in situations where camera vision sensors for lane recognition are unavailable. RL is aimed at maximizing a reward function through trial and error, based on observed data. As shown in Figure 3, surrounding object information undergoes postprocessing and is then transmitted to the RL model, which generates appropriate actions. The postprocessing stage includes the transformation of data from global coordinates to local coordinates and filtering of nearby vehicles from the entire set of vehicles. Real-time data from the CarMaker simulation are acquired using the CM read utility, including coordinates, velocity, acceleration, and angles of surrounding objects, all provided in global coordinates with respect to the road. Subsequently, these data are converted into the local coordinates for LKS control and filtering is performed to select the closest five vehicles within the specified range of the target vehicle to be controlled.

### 3.2. Training Environment

Vehicle simulation programs must adhere to certain crucial requirements to ensure that the simulation environment mimics real vehicle conditions. In particular, any discrepancies between the simulation and real environments can render the results inconclusive. Therefore, it is essential to configure a simulation environment that takes into account the dynamic elements of actual vehicles. Additionally, the simulation must be capable of being executed at speeds exceeding real-time requirements, because RL involves repeating scenarios and learning through trial and error. Compatibility with other software programs is also a key consideration, as it may be necessary to integrate the data generated within the vehicle simulation environment with other programs for training [16]. In this study, we satisfy these requirements by configuring the learning environment using the IPG CarMaker program version 11.1 and Matlab 2021a, ensuring seamless integration and compatibility with other software programs. This platform enables real-time monitoring of data during testing and faithful replication of realistic vehicle behavior through parameterized components, such as steering, tires, brakes, powertrain, and chassis. Moreover, it supports Simulink integration, enabling data analysis [32]. The DDPG RL agent is implemented using MATLAB Simulink, with the hyperparameters set as outlined in Table 1 to configure the learning environment.

### 3.3. DDPG Algorithm

The DDPG RL algorithm has demonstrated its potential in the field of control by outperforming traditional path control methods when applied to vehicles following predefined paths [33]. The DDPG algorithm employs the actor–critic network framework to update the actor and critic models [34]. Perform the training steps in the following sequence.

1. Randomly initialize the critic Q(S,A;∅) and actor π(S;θ) with parameter values $\emptyset_{t}$ and $\theta_{t}$, respectively.
2. Utilize Equation (1) to determine the action to take based on the current observation S, where *N* represents the stochastic noise component of the noise model defined via NoiseOptions:
  (1) $$A=\pi \left(S;\theta \right)+N.$$
3. Take action *A*, and then observe the reward *R* and subsequent observation *S′*.
4. Store the experience (*S*,*A*,*R*,*S*′) in the experience buffer.
5. Randomly select a mini-batch of M experiences ($S_{i},A_{i},R_{i},S_{i}^{\prime }$) from the experience buffer, where *M* is determined by the value assigned to the MiniBatchSize option.
6. If $S_{i}^{\prime }$ is a terminal state, set the value function target *y_i_* to *R_i_*:
  (2) $$y_{i}=R_{i}+\gamma Q^{\prime }(S_{i}^{\prime },u^{\prime }(S_{i}^{\prime }|\theta_{u})|\theta_{Q}^{\prime }).$$
7. Optimize the critic parameters by minimizing the loss L calculated using Equation (3) over all the sampled experiences:
  (3) $$L=\frac{1}{M}\sum_{i=1}^{M}{{(y}_{i}-Q(S_{i,}A_{i}|\theta_{Q}))}^{2}.$$
8. Optimize the actor parameters using Equation (4):
  (4) $$\nabla \theta_{u}J\approx \frac{1}{M}\sum_{i=1}^{M}G_{ai}G_{ui},\;G_{ai}=\nabla_{A}Q\left(S_{i},A|\theta_{Q}\right)where\;A=u\left(S_{i}|\theta_{u}\right),\;G_{ui}={\nabla \theta }_{u}u\left(S_{i}|\theta_{u}\right).$$
9. Update the target actor and critic parameters depending on the target update method.

### 3.4. DDPG Actor–Critic Network

In this study, the network layers are configured as indicated in Table 2 and Table 3. The network architecture consists of actor and critic networks, each including a feature input layer, fully connected layers with nodes, and rectified linear unit (ReLU) activation functions. In the actor network, the final steering value is output by the fully connected layer connected to a tanh activation function. The scaling layer is used to determine the control range. This design enables the mapping of the actor’s output to the desired steering control within the specified range.

### 3.5. State Space

**Figure 4:**
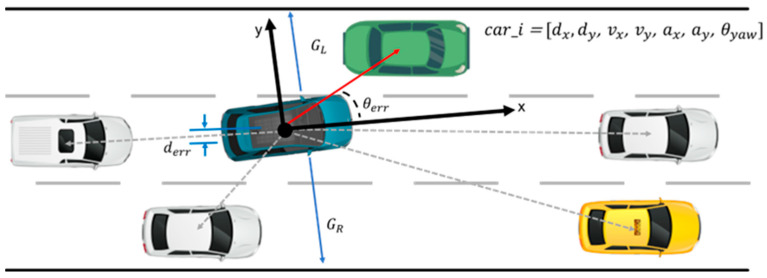
State space of the RL environment.

The state space represents the observable values in the environment. In this study, the state space encompasses all three elements used in the training environment, as shown in Figure 4, which includes the observation space, reward, and termination states. We have defined the state space in Table 4. The information about surrounding vehicles focuses on the closest five vehicles.

### 3.6. Observation Space

Observation space refers to the values the agent perceives in the environment. This is different from the state space. In this study, we train the LKS steering using only information from surrounding static and dynamic objects. The observation space includes information about the surrounding vehicles and the distance to the guardrail. The guardrail information is included only in the guardrail scenario and not in the basic or Gaussian noise scenarios. We define the observation space in Table 5.

**Figure 5:**
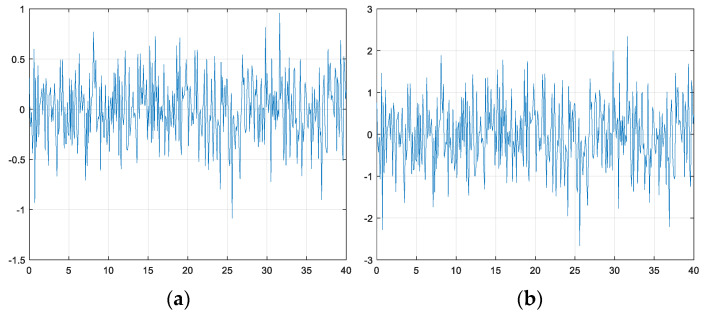
(**a**) Position Gaussian noise (Variance, 0.1). (**b**) Velocity Gaussian noise (Variance, 0.6). Gaussian noise to mimic real sensor noise.

To better emulate real-world conditions, Gaussian sensor noise is introduced to the position and velocity values, as shown in Figure 5. This addition of sensor noise is essential for assessing the direct applicability of the results to real vehicles. Specifically, the addition of noise renders the simulation more realistic, accounting for the uncertainty and variability that real-world sensors typically exhibit. The testing scenarios in this study involve single-lane and four-lane roads. Because only nearby vehicle information is required to maintain the lane, the forward and rear vehicle perception ranges are set as 40 m and 10 m, respectively. A maximum of five vehicles are considered in the perception range. The state values of these vehicles are summarized in Table 4. The state values of vehicles beyond the perception range are assumed to be 0.

### 3.7. Action Space

The action space is structured for lateral control of the vehicle, specifically in terms of the steering angle, as presented in Table 6. Longitudinal control is managed by setting a default target speed of 50 kph, controlled by a PI controller based on the internal parameters of CarMaker. CarMaker provides an adaptive cruise control (ACC) controller, which functions by maintaining the target speed when no leading vehicle is detected, and it engages the ACC feature when a leading vehicle is detected. The controller is composed of a PI controller and adjusts the position of the brake and gas pedals to control the longitudinal acceleration. Additionally, it includes a speed adjustment feature based on the road curvature. The rate of change of the steering angle for the lateral control is limited to a maximum of ±150°/s to prevent abrupt steering actions, thereby ensuring stable control performance. The range of steering wheel control angles is confined within −180° to +180°. These constraints are designed to maintain safe and stable control of the vehicle during the learning process.

### 3.8. Reward Function

The reward function calculates rewards during the learning process based on how well the agent’s actions align with the intended objectives. It provides feedback to the agent to guide it toward learning optimal behaviors. In this study, a reward function is designed to address lane-keeping control problems using RL. This function encompasses terms related to lane-keeping, collision avoidance, and steering wheel oscillation prevention, as shown in Equation (5). This function plays a pivotal role in shaping the agent’s behavior by assigning rewards for actions that contribute positively to the desired objectives while penalizing actions that lead to deviations.

(5) $$R\left(v_{x},\theta_{err},d_{err},\delta ,T_{s}\right)=\;k_{1}\left|v_{x}cos\theta_{err}\right|+k_{2}\left|v_{x}sin\theta_{err}\right|+k_{3}\left|d_{err}\right|+k_{4}\left|\delta_{t}-\delta_{t-1}\right|+k_{5}\left|T_{s}\right|$$

The reward function weights for training are shown in Table 7. $k_{1}$ governs the preview error for lane-keeping control, while $k_{2}\;and\;k_{3}$ are negative compensatory values to minimize lateral deviation of the vehicle. $k_{4}$ is used to mitigate steering oscillations. This parameter is essential in preventing unstable control behavior that may arise if the sole objective is lane-keeping. $k_{5}$ enhances the simulation robustness and encourages the expansion of simulations.

### 3.9. Termination States

The definition of appropriate termination conditions is crucial in RL to ensure that the learning process converges effectively and can prevent unpredictable or undesirable behaviors. Without well-defined termination conditions, an RL agent might continue learning indefinitely, which can lead to inefficient learning and potentially harmful behaviors. The learning process terminates when the agent achieves satisfactory performance. If intervals of decreasing learning performance are detected thereafter, the training is halted. The satisfaction score is based on the reward function applied when reinforcing the CarMaker controller with reinforcement learning. During this process, an agent that performed well in the interval from the steady state until a decline in scores is selected. For this research, the following two termination conditions are defined:

- If the ego vehicle deviates from the intended path or angle by more than a specified threshold:
  ($\left|d\right|\geq d_{max}\;or\;\left|\theta \right|\geq \theta_{max}$).
- If there is a collision between the ego vehicle and surrounding vehicles or obstacles:
  (if Collision Signal=1).
- If the learning score meets the satisfactory performance criterion and intervals of performance decline are identified afterward.

Should either of these conditions be met, the ego vehicle within the simulation is directed to return to the starting point and initiate a new episode.

## 4. Results

**Figure 6:**
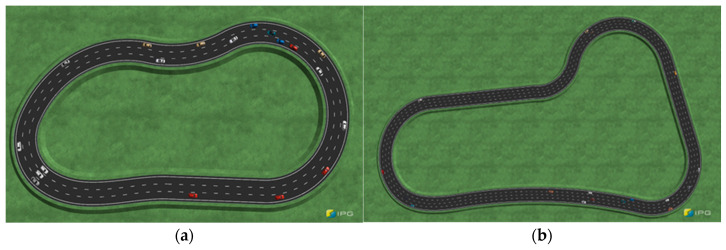
(**a**) Road for the first scenario. (**b**) Road for the second scenario. CarMaker roads for assessing lane-keeping system control performance.

The objective of the proposed approach is to prevent accidents in the event of a camera vision sensor failure by providing a fault-tolerant system for sensor malfunctions. To assess the performance of lane-keeping control based on surrounding vehicle information without the use of vision sensors for lane recognition, two scenarios are designed, and simulations are conducted. In the simulation environment, elements such as the vehicle’s target speed, the number of vehicles on the road, and road friction, are the same, but there are differences in the movement of surrounding vehicles. The agent controls the vehicle by following the steering values generated while maintaining the lane. The learning process is initiated at a time step of 10 Hz. As shown in Figure 6, the two scenarios, with durations of 40 s and 60 s, are implemented, corresponding to 400 and 600 steps, respectively.

### 4.1. Simulation Scenario

**Figure 7:**
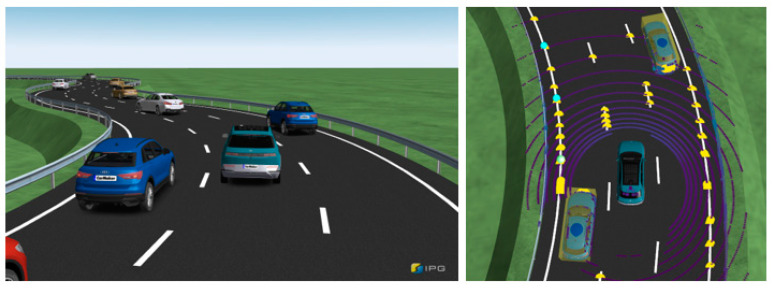
IPG movie displaying the first scenario road and surrounding vehicles.

The first validation scenario involves a single three-lane road, as shown in Figure 7, with a driving scenario lasting approximately 40 s. In the RL process, the agent is trained using observations of the relative positions, relative velocities, relative accelerations, and relative heading angles of the surrounding vehicles.

The performance is validated through three tests: the basic case, which involves only the surrounding object information; the guard rail case, which includes information regarding guard rail distances; and the noise case, in which Gaussian sensor noise is introduced. The ego vehicle operates at 50 kph using CarMaker internal PI controller and functions with the ACC system. Additionally, the surrounding vehicles perform cut-in and cut-out instead of maintaining their current lane. Cut-in and cut-out of surrounding vehicles are made by autonomous settings of the traffic maneuver and occur randomly.

**Figure 8:**
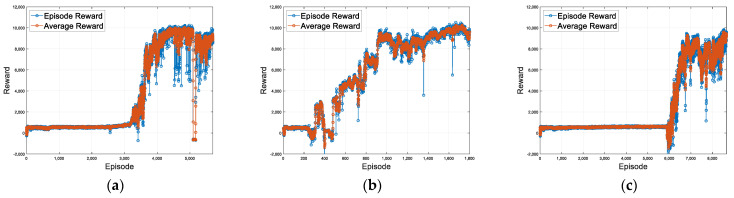
(**a**) First scenario basic case training reward. (**b**) First scenario guard rail case training reward. (**c**) First scenario noise case training reward. Training reward plots for the first scenario RL.

**Figure 9:**
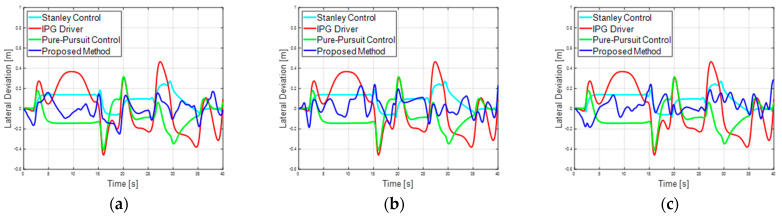
(**a**) First scenario basic case lateral deviation performance. (**b**) First scenario guard rail lateral deviation performance. (**c**) First scenario noise case lateral deviation performance. Comparison plots of model-based controller and proposed method for reinforcement learning-based lateral tracking performance.

Figure 8 shows the increase in reward values as learning progresses and episodes accumulate. The horizontal axis of the graph represents the episode count, where each episode corresponds to the simulation starting and ending via termination conditions. The vertical axis represents the rewards, indicating the cumulative reward values for each episode. When compared to the basic case, the guardrail case with added guardrail information showed the fastest and highest performance during learning. In contrast, the Gaussian case demonstrated slower learning progress due to the noise. In the first scenario, the basic, guard rail, and noise cases demonstrate successful completion of the course without any collisions in the initial 40 s, starting from episodes 3415, 509, and 6185, respectively. Figure 9 shows the lane center tracking performance of the proposed method for lane maintenance compared with those of three lateral control methods: the pure pursuit controller, Stanley controller, and IPG driver. In the graph, the horizontal axis represents simulation time, while the vertical axis represents lateral deviation. The proposed method, which is based on RL learning using information from surrounding vehicles, demonstrates performance similar to a traditional controller such as a pure pursuit or Stanley controller that operates with knowledge of lane information. At a target speed of 50 kph, maximum lateral errors of 0.2507 m, 0.2387 m, and 0.2850 m are observed in the basic, guard rail, and noise cases, respectively. This result showcases that the proposed approach can effectively perform lateral control for autonomous vehicles using information from surrounding vehicles.

**Figure 10:**
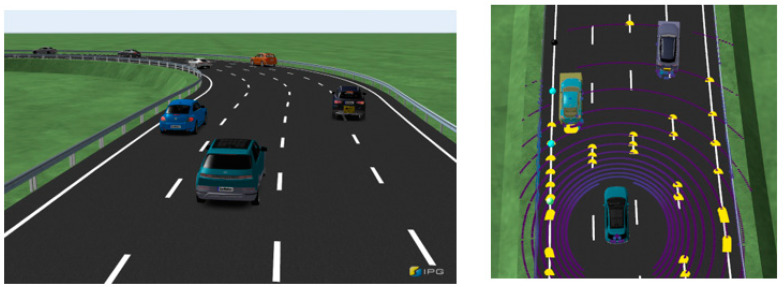
IPG movie displaying the second scenario road and surrounding vehicles.

The second validation scenario involves a single four-lane road with a driving duration of approximately 60 s, as shown in Figure 10. Similar to the previous scenario, the agent learns from observations including the relative position, relative speed, relative acceleration, and relative heading angle. The simulation cases are categorized into three scenarios: basic, guard rail, and gaussian cases. The difference between the first and the second scenarios lies in the fact that the second scenario features a single four-lane road, resulting in sparser traffic within the perception range and a higher frequency of surrounding vehicles performing cut-ins and cut-outs. This scenario poses a more challenging problem for learning within the training environment compared to the previous scenario.

**Figure 11:**
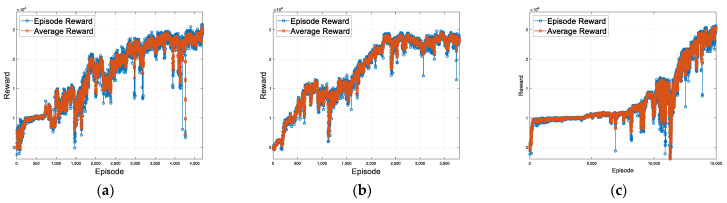
(**a**) Second scenario basic case training reward, (**b**) Second scenario guard rail case training reward. (**c**) Second scenario noise case training reward. Training reward plots for the second scenario RL.

**Figure 12:**
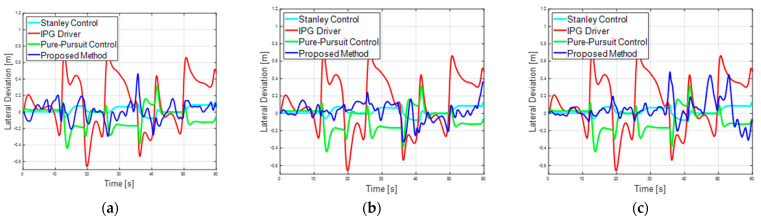
(**a**) Second scenario basic case lateral deviation performance. (**b**) Second scenario guard rail lateral deviation performance. (**c**) second scenario noise case Lateral deviation performance. Comparison plots of model-based controller and proposed method for reinforcement learning-based lateral tracking performance.

Figure 11 depicts the reward graph based on episodes for the second scenario. The horizontal axis represents the episode count, while the vertical axis represents the rewards, signifying the cumulative reward values at each episode. Similar to the previous first scenario, the guard rail case demonstrated the fastest and highest performance, whereas the Gaussian case exhibited lower performance. In the driving scenarios, the vehicle speed varies according to the curvature of the road, deviating from a constant speed profile. Starting from episodes 2377, 1585, and 11,236 for the basic case, guard rail case, and noise case, respectively, the course is successfully completed over 60 s without any collisions. The maximum lateral deviation for the basic case, guard rail case, and noise case are 0.4641 m, 0.3617 m, and 0.4764 m, and the RMS errors are 0.1121, 0.1067, and 0.1412, respectively. Figure 12 compares the performance of the proposed method with three other lateral control methods, highlighting the lane center tracking performance of the proposed method for lane maintenance. The horizontal axis of the graph represents time, while the vertical axis shows the lateral deviation. In the second scenario, the proposed method demonstrates performance similar to a traditional controller such as a pure pursuit controller.

### 4.2. Proposed Method for Fault-Tolerant System Using RL

We have developed a fault-tolerant system that allows autonomous vehicles (AVs) to perform a lane-keeping system (LKS) based on LiDAR sensor-derived surrounding object recognition information in the event of camera sensor failures in lane recognition. Table 8 demonstrates the control performance through reinforcement learning based on the observation space information explained in Section 3.6. The scenarios are divided into two categories, and each scenario underwent testing under three different cases: basic, guard rail, and Gaussian. The basic case relies on surrounding vehicle information, excluding guard rail distances, as presented in Table 5, for navigation. The guard rail case involves adding guard rail information to the basic case. The Gaussian case maintains the same observation configuration as the basic case but introduces Gaussian noise to the surrounding object information. The objective of our research is to evaluate the performance in terms of the lateral deviation of the control vehicle for the implementation of the LKS driving within the fault-tolerant system (FTS). As seen in Table 8, the performance appears to be better in the order of guard rail, basic, and Gaussian noise cases. Additionally, scenario 1 outperformed scenario 2, which presents a more challenging problem. Nevertheless, all three cases demonstrated the ability to safely maintain the lane without collisions or lane departures during the simulation period. In addition, to ensure the safety of the system, we conducted additional testing with 100 different starting positions for the Ego vehicle in each simulation. In all 100 cases, the vehicle successfully completed the scenarios without any collisions. The scenarios were designed to include challenging conditions such as various curves, stationary vehicles, and lane-changing vehicles, demonstrating the robustness of the system in adverse environments. The successful completion of these additional 100 tests further confirms the safety assurance of the FTS.

### 4.3. Comparison with Traditional Controllers

We conducted a comparative analysis of the performance of the proposed method, which is based on lane information for lateral control, with the pure pursuit, Stanley controller, and IPG Driver. Pure pursuit and Stanley controllers are widely used in autonomous driving control and have proven performance. The chosen criteria for comparison are lateral deviation and heading angle error. These two factors play a crucial role in optimal lateral control for autonomous driving. Traditional optimal control aims to minimize the errors in these two factors, making a comparison of their performance meaningful in the context of this study as shown in Table 9 and Table 10. The IPG Driver is a control method provided by CarMaker, based on the pure pursuit model, and it reflects human driving characteristics. When comparing the performance of the proposed method with the guard rail case, in scenario 1, it exhibited better performance in terms of maximum deviation and RMS for the entire segment, outperforming pure pursuit and IPG Driver. In scenario 2, it showed lower maximum deviation and better overall performance than the IPG Driver. As shown in Table 9 and Table 10, the proposed method demonstrated performance similar to that of traditional controllers, indicating its ability to reliably establish a fault-tolerant System. When comparing the results of learning the guard rail scenario using TD3, a more recent algorithm, the target score was achieved. Although there is a difference in the tendency of reward, it was confirmed that a similar performance is demonstrated once the steady state is reached.

## 5. Conclusions and Future Work

In this paper, we have presented research on a reinforcement learning (RL)-based fault-tolerant system. The agent’s input observation includes information on a maximum of five surrounding vehicles within the perception range, and in specific cases, the distance to guardrails is also added. The output is steering commands. The key aspect of this approach lies in its ability to address situations not only involving the failure of vision sensors, such as cameras for lane recognition, but also situations involving lane damage. The reward function of the proposed method is composed of elements for vehicle lane center tracking to facilitate LKS. Additionally, elements related to steering oscillation are included to enhance steering safety and reduce the computational cost of actions. Even when comparing the performance of learning in scenarios where lane information is known to traditional control methods like pure pursuit and Stanley, our method demonstrates sufficient performance for LKS functionality. While the research presented in this paper has shown promising results, there is room for improvement in terms of learning performance and safety. In this study, we employed the deep deterministic policy gradient (DDPG) reinforcement learning algorithm, which is widely used in lateral control. To address the limitations of the DDPG algorithm, which is advantageous for continuous control, new algorithms like twin delayed deep deterministic policy gradient (TD3) and soft actor-critic (SAC) have been developed to provide more stable learning. Currently, efforts are underway to establish the SAC environment to assess the learning trends. Furthermore, it is important to note that autonomous driving using deep learning faces challenges in terms of debugging, and there is an ongoing need to establish methods to achieve safety standards, as regulations like Safety Of The Intended Functionality (SOTIF): ISO/PAS 21448:2019 are not yet fully established. In future research, we plan to apply state-of-the-art reinforcement learning algorithms that take into account dynamic factors such as lateral acceleration, jerk, and side-slip angle. We intend to train an agent capable of simultaneous lateral and longitudinal control of the vehicle.

## Figures and Tables

**Table 1:** Hyperparameters for DDPG model training.

Parameter	Value
Discount factor (*γ*)	0.99
Target smooth factor	0.001
Mini-batch size	64
Target network update frequency	100
Replay memory size	10^7^
Noise variance	0.6
Noise variance decay rate	10^6^

**Table 2:** DDPG actor network structure.

	Neurons	Name
Feature input layer	35, 37	Observation
Fully connected layer	100	ActorFC1
Rectified linear unit		Relu1
Fully connected layer	100	ActorFC2
Rectified linear unit		Relu2
Fully connected layer	100	ActorFC3
Rectified linear unit		Relu3
Fully connected layer	1	ActorFC4
Hyperbolic tangent layer		Than1
Scaling layer		Actorscaling1

**Table 3:** DDPG critic network structure.

State Path		
	Neurons	Name
Feature input layer	35, 37	Observation
Fully connected layer	100	CriticFC1
Rectified linear unit		Relu1
Fully connected layer	100	CriticFC2
Addition layer	2	Add
Rectified linear unit		Relu2
Fully connected layer	1	CriticFC3
Action path		
Feature input layer	1	Action
Fully connected layer	100	CriticActionFC1

**Table 4:** State space definitions for RL training.

State (i = 0,1,2,3,4)	Meaning
$S_{7i+1},S_{7i+2}$	Object relative distance, $d_{x,}d_{y}$
$S_{7i+3},S_{7i+4}$	Object relative velocity, $v_{x,}v_{y}$
$S_{7i+5},S_{7i+6}$	Object relative acceleration, $a_{x,}a_{y}$
$S_{7i+7}$	Object relative heading, $\theta_{yaw}$
*S*_36_	Ego vehicle lateral offset, $d_{err}$
*S*_37_	Ego vehicle heading offset, $\theta_{err}$
*S*_38_	Collision status
*S*_39_	Longitudinal velocity, $v_{x}$
*S*_40_	Simulation time, $T_{s}$
*S*_41_	Steering angle, δ
*S*_42_,*S*_43_	Guard rail distance, $G_{L,}G_{R}$

**Table 5:** Observation space definitions for RL training.

Observation (i = 0,1,2,3,4)	Meaning
$O_{7i+1},O_{7i+2}$	Object relative distance, $d_{x,}d_{y}$
$O_{7i+3},O_{7i+4}$	Object relative velocity, $v_{x,}v_{y}$
$O_{7i+5},O_{7i+6}$	Object relative acceleration, $a_{x,}a_{y}$
$O_{7i+7}$	Object relative heading angle, $\theta_{yaw}$
$O_{36},O_{37}$	Guard rail distance, $G_{L,}G_{R}$

**Table 6:** Action space for LKS control. The steering angle is adjustable within the range of −180° to 180°.

State	Description	Min	Max
*δ*	Steering angle	−180°	180°

**Table 7:** Reward function weights for training.

*k*_1_	*k*_2_	*k*_3_	*k*_4_	*k*_5_
20	1	40	1	300

**Table 8:** Lane centering performance in terms of maximum lateral deviation and RMS error for the entire segment.

Proposed Method RL Controller	Scenario 1		Scenario 2	
	Max_Deviation [m]	Deviation RMS	Max_Deviation [m]	Deviation RMS
Basic	0.2507	0.0904	0.4641	0.1121
Guard rail	0.2387	0.0889	0.3617	0.1067
Gaussian noise	0.2850	0.0908	0.4764	0.1412

**Table 9:** Comparison of lane centering performance between the proposed method and traditional controller.

Controller		Scenario 1		Scenario 2	
		Max_Deviation [m]	RMS Error	Max_Deviation [m]	RMS Error
Traditional Control	Pure pursuit	0.3154	0.1487	0.3149	0.1398
	Stanley	0.2716	0.1151	0.1308	0.0494
	IPG Driver	0.4646	0.2376	0.7095	0.3398
RL Control	TD3	0.2421	0.0903	0.3648	0.1147
	Ours	0.2387	0.0889	0.3617	0.1067

**Table 10:** Comparison of heading error between the proposed method and traditional controller.

Controller		Scenario 1		Scenario 2	
		Max_Heading [deg]	RMS Error	Max_Heading [deg]	RMS Error
Traditional Control	Pure pursuit	3.9086	3.1578	4.7712	1.9117
	Stanley	2.3507	2.2792	3.1480	1.7052
	IPG Driver	4.7928	3.2544	6.7176	2.1671
RL Control	TD3	2.3418	2.1798	4.8529	1.8653
	Ours	2.2531	2.1546	4.7953	1.8295

## Data Availability

Data are contained within the article.
